# How digital interventions on screening and BI might be applied to psychiatric ED settings

**DOI:** 10.1186/1940-0640-10-S2-O10

**Published:** 2015-09-24

**Authors:** Frederic C Blow, Kristen Lawton Barry

**Affiliations:** 1 Center for Clinical Management Research, Ann Arbor Veterans Affairs Healthcare System, Ann Arbor, Michigan, 48109, USA; 2 Addiction Center, Department of Psychiatry, University of Michigan, Ann Arbor, Michigan, 48109, USA; 3 Institute for Healthcare Policy and Innovation, University of Michigan, Ann Arbor, Michigan, 48109, USA

## Background

Unhealthy drinking and other drug use are common comorbid problems among individuals receiving treatment in psychiatric Emergency Departments (ED). Several studies have shown that systematic screening and motivational brief interventions (SBIRT) are promising approaches for reducing alcohol and drug use that can be implemented in psychiatric ED settings. Recent digital screening and brief intervention trials have shown reductions in alcohol and other drug use among general (non-psychiatric) ED patients. These electronic approaches have yet to be tested for those receiving care in psychiatric ED settings. This presentation will provide an overview and framework for considering how SBIRT might be delivered electronically among psychiatric ED patients and how digital devices might be used to extend the impact of SBIRT beyond the psychiatric ED.

## Methods

Systematic literature review of published literature on digital behavioral interventions for individuals with major mental disorders. Review of existing literature on electronic SBIRT approaches in the ED setting.

## Results

Several studies have shown promising results for the use of tablet computers, wearable tracking devices and smartphone apps for individuals with mental disorders. The targeted behavioral interventions have typically focused on general fitness and weight loss. Several published studies have shown promise for digital SBIRT and computer-assisted SBIRT approaches (computer decision support) addressing alcohol and other drug use in *general* ED and other healthcare settings. However, the potential use of digital interventions for alcohol and other drug use among *psychiatric* ED patients have been inadequately explored.

## Conclusions

Integrating digital SBIRT interventions initiated in psychiatric ED settings, with potential continuing care using mobile devices, could both improve patient access to SBIRT and provide alternative enhancements to psychiatric care to improve outcomes for these vulnerable patients. Electronic delivery of SBIRT, combined with the use of other digital technologies for patients with major mental disorders and concurrent substance misuse, should be explored.

